# Tele-Active Rehabilitation for Youth With Concussion: Evidence-Based and Theory-Informed Intervention Development

**DOI:** 10.2196/34822

**Published:** 2022-04-04

**Authors:** Josh Shore, Emily Nalder, Michael Hutchison, Nick Reed, Anne Hunt

**Affiliations:** 1 Rehabilitation Sciences Institute University of Toronto Toronto, ON Canada; 2 Department of Occupational Science and Occupational Therapy University of Toronto Toronto, ON Canada; 3 Faculty of Kinesiology and Physical Education University of Toronto Toronto, ON Canada

**Keywords:** concussion, pediatrics, active rehabilitation, telehealth, exercise, mobile phone

## Abstract

**Background:**

Active rehabilitation involving subsymptom threshold exercise combined with education and support promotes recovery in youth with concussion but is typically delivered in person, which may limit accessibility for families because of a lack of services in their communities or logistical challenges to attending in-person sessions.

**Objective:**

This paper describes the evidence-based and theory-informed development of the Tele-Active Rehabilitation (Tele-AR) intervention for pediatric concussion, which was specifically designed for remote service delivery.

**Methods:**

The intervention was designed by clinician-researchers with experience in pediatric concussion rehabilitation following the Medical Research Council guidance for developing complex interventions. Development involved a critical review of the literature to identify existing evidence, the expansion of the theoretical basis for active rehabilitation, and the modeling of the intervention process and outcomes.

**Results:**

Tele-AR is a 6-week home exercise and education and support program facilitated through weekly videoconferencing appointments with a clinician. Exercise consists of low- to moderate-intensity subsymptom threshold aerobic activity and coordination drills that are individualized to participant needs and interests (prescribed for 3 days per week). Education includes the evidence-supported *Concussion & You* self-management program, which covers topics related to energy management, nutrition, hydration, sleep hygiene, and return to activity. Elements of self-determination theory are incorporated to support motivation and engagement. We present a logic model describing predicted intervention effects using a biopsychosocial conceptualization of outcomes after concussion.

**Conclusions:**

The Tele-AR intervention may help to increase access to care that improves recovery and promotes a timely return to activity in youth with concussion. Future research is needed to evaluate the feasibility and efficacy of this approach.

## Introduction

### Overview

Concussion is a common injury among children and adolescents that may result in physical, cognitive, emotional, or sleep-related symptoms [1-3]. Although many youth with concussion achieve symptom resolution within 2 to 4 weeks after injury, approximately 30% continue to experience persistent symptoms beyond 4 weeks [3]. Youth experiencing persistent concussion symptoms report lower quality of life [4] and higher incidence of mental health disturbances [5] than noninjured peers.

### Occupational Performance and Participation in Youth With Concussion

Occupational performance, which refers to the ability to choose, organize, and satisfactorily perform meaningful occupations [6], is often impaired in youth with concussion [7]. Qualitative studies highlight that concussion symptoms limit performance in meaningful activities such as school, physical activity or sport, and social activities [8,9]. Youth with concussion report diverse occupational performance issues in the domains of self-care, productivity, and leisure that can be improved after rehabilitation, underscoring the utility of occupational performance as a measure of functional recovery [7]. Youth may also experience limitations in participation, defined as involvement in a life situation [10]. Although activities and participation generally improve within the first few months after concussion, a substantial portion (30%-60%) of youth continue to report lower participation than their peers at 6 months after injury [10]. Continued limitations in activities and participation may lead to physical deconditioning or threats to psychological well-being, including anxiety, depression, and social isolation [11,12].

Currently, recovery from concussion among children and adolescents is being reconceptualized to emphasize resumption of everyday activities and general well-being, rather than solely the resolution of postconcussion symptoms [13]. Evaluating occupational performance can help to identify limitations and inform active rehabilitation strategies to support youth in gradual resumption of meaningful activities, thus decreasing the impact of the injury and reducing risk for persistent sequelae [14,15].

### Active Rehabilitation for Youth With Concussion

Growing evidence supports an active approach to concussion rehabilitation involving guided physical activity. Several studies demonstrate the efficacy of subsymptom threshold aerobic exercise for reducing postconcussion symptoms [16-24]. Some active rehabilitation programs for youth have also included other components such as sport-specific coordination drills [25-31], balance training [32], visualization and relaxation [25-31], and education and support [25-31]. Results from these studies show that active rehabilitation reduces postconcussion symptoms and improves recovery [25-32].

The rationale for an active approach to concussion rehabilitation has been rooted in the benefits of physical exercise to promote biological recovery (ie, improve cerebrovascular autoregulation and increase neuroplasticity) and psychological well-being (ie, increase self-efficacy and improve mood) [28,33-35]. To date, outcome measurement has predominantly focused on changes in postconcussion symptoms [36]. However, evidence indicates that although concussion symptoms initially arise because of physiological changes, including metabolic and cerebral autoregulatory dysfunction, the strength of the relationship between the neurobiology of the injury and ongoing symptom experience diminishes over time, with noninjury factors playing an important role in the persistence of symptoms [37]. Recovery from concussion is therefore best understood through a biopsychosocial model in which psychological and social factors, including activity restriction, emotional reaction, and coping styles, interact with biological factors to maintain symptoms and functional limitation after concussion [13,38-40]. Accordingly, an active rehabilitation approach should aim to restore performance in daily activities in addition to reducing postconcussion symptoms.

The theoretical model of active rehabilitation described by Gagnon et al [28] endorses a biopsychosocial perspective by emphasizing the physiological, psychological, and social benefits of controlled exercise in combination with education and motivation. However, although the biopsychosocial model helps to identify targets for intervention, it does not address how to support motivation, which is an important determinant of outcomes in pediatric rehabilitation [41] and is often reduced after brain injury [42]. Motivation is particularly important during active rehabilitation because the intervention typically involves a home program that youth complete independently. Extending the theoretical rationale for active rehabilitation could help to guide how to promote motivation and further address the impact of such interventions on performance of daily activities and general well-being.

### Barriers to Accessing Active Rehabilitation

To date, active rehabilitation has typically been conducted through in-person sessions at a specialty clinic or research facility [27,28,30,31]. However, access to specialty health care is often limited for youth in Canada because of a lack of specialty services in their geographic region, as well as financial and logistical barriers to parental accompaniment [43,44]. Telerehabilitation has emerged as an effective method of service delivery that may address challenges in access. In a systematic review, clinical outcomes from telerehabilitation were found to be similar to, or better than, those from traditional in-person interventions for a variety of conditions [45]. Measures of clinical process, including attendance, compliance, and satisfaction, were also generally high [45]. Among youth, reviews highlight telerehabilitation as an appropriate method of service delivery [46,47] that may be especially effective when targeting behavioral function using a coaching approach and exercise program [46]. The role of telemedicine in concussion care is currently expanding [48,49] but has yet to be explored in the delivery of an active rehabilitation intervention.

This paper describes the evidence-based development of, and theoretical rationale for, the Tele-Active Rehabilitation (Tele-AR) intervention for youth with concussion, which was specifically designed to promote return to activity in a remote service delivery format. A detailed overview of the intervention is also provided.

## Methods

### Ethical Approval

This study was approved by the University of Toronto Health Sciences Research Ethics Board (REB reference number 00039179). Participants provided informed written consent to participate in the study.

### Intervention Development Process

The Tele-AR intervention was designed by a team of clinician-researchers (kinesiologists and occupational therapists) with experience in pediatric concussion rehabilitation, based on clinical experience and a critical review of published literature. The Medical Research Council (MRC) framework for developing and evaluating complex interventions [50,51] guided the development process.

According to the MRC framework, intervention development is an iterative process involving integration of current evidence and appropriate theory, followed by a phased testing approach involving a series of pilot studies before a definitive evaluation [50]. The development stage involves the following three key processes: (1) identifying the existing evidence base, (2) identifying or developing appropriate theory, and (3) modeling process and outcomes.

### Key Tasks

We drew on guidance from Campbell et al [52] and Faes et al [53] regarding key tasks for each of the MRC development processes. According to Campbell et al [52], existing evidence should be used to develop an understanding of the problem and target context to identify opportunities for intervention. Key tasks include defining and quantifying the target population most likely to benefit, defining the health outcome and appropriate measures, understanding pathways that cause and sustain the problem, identifying similar interventions, and predicting barriers or design challenges [53]. Integrating appropriate theory involves specifying theory-based determinants of change and describing how the intervention will affect them [53]. Finally, the development of a conceptual or logic model is recommended to define intervention components and clarify anticipated mechanisms linking components to desired targets for change [52]. Textbox 1 shows the application of the MRC framework and key tasks to development of the Tele-AR intervention.

**Application of key tasks from the Medical Research Council framework to the development of the Tele-Active Rehabilitation intervention.**

**Identifying existing evidence**

- Define and quantify target population
  Review of concussion epidemiology among youth
  Project team consultation to determine target population
- Define health outcome and outcome measures
  Review of current consensus definition of recovery from concussion
  Identification of appropriate measurement tools
- Understand factors that cause and sustain the problem
  Review of factors that influence concussion recovery in youth
  Identification of existing biopsychosocial models of concussion
- Identify similar interventions
  Critical review of literature pertaining to active rehabilitation interventions for youth with concussion
  Review of best practice recommendations for telerehabilitation and telehealth self-management interventions
- Define target context
  Review of guidelines for telemedicine in concussion management

**Identifying appropriate theory**

- Review of existing theoretical rationales for active rehabilitation
- Review of health behavior change theories
- Integration of health behavior change theory in the intervention

**Modeling process and outcomes**

- Project team consultation to design intervention components
- Development of logic model to describe anticipated effects of intervention components

## Results

### Developing the Intervention

Results from the development stage are described in the following sections, organized according to the MRC headings and key tasks identified in the *Methods* section.

### Identifying Existing Evidence

#### Define and Quantify Target Population

The Tele-AR intervention targets adolescents (aged 13-17 years) who continue to experience symptoms at least 2 weeks after concussion. All mechanisms of injury are considered, including sport, falls, and motor vehicle accidents.

In recent decades, health care usage for pediatric concussion has increased substantially in Canada [1] and the United States [54]. In Ontario, this increase has been most pronounced among adolescents [1], for whom the average annual incidence of physician-diagnosed concussion was approximately 1500 per 100,000 from 2008 to 2016 [55]. Adolescents are also known to be at high risk for prolonged recovery [56]. According to a Canadian multicenter prospective study, a substantial subset of adolescents, approximately 30%, continue to experience symptoms beyond 1 month after concussion [3], which may lead to mood disturbances [5], reduced quality of life [4], and impaired activity performance [7]. Results from the same multicenter study also indicate that symptom improvement occurs primarily within the first 2 weeks of injury and levels off 2 to 4 weeks after injury [57]. Exercise-based active rehabilitation programs initiated in the subacute phase of recovery (1-2 weeks after injury) are feasible and may reduce the risk of persistent symptoms [19,20,24,26]. The Tele-AR intervention therefore targets adolescents continuing to experience symptoms beyond 2 weeks after concussion who are at risk for developing secondary consequences related to mood, activity performance, and quality of life.

#### Define Health Outcome and Outcome Measures

Primary health outcomes for the Tele-AR intervention include postconcussion symptoms and perceived occupational performance, a measure of performance in daily activities.

The most recent (2016) international consensus statement on sports-related concussion defines recovery as the resolution of concussion symptoms and return to normal activities, including school, work, and sport [2]. Self-reported concussion symptoms remain the most common measure of recovery from concussion [58] and are measured in adolescents using an age-appropriate symptom scale such as the Postconcussion Symptom Inventory [59]. However, there is growing recognition that symptom reports do not always accurately reflect recovery [13]. Concussion-like symptoms (eg, fatigue, headache, and dizziness) are common in individuals without a history of concussion, and symptom reporting is influenced by a variety of noninjury factors such as personality, comorbid mood disorders, and familial factors [12]. Activity limitations are common among adolescents with concussion [7-9]; therefore, a measure of performance in daily activities (ie, occupational performance) is beneficial for evaluating recovery. The Canadian Occupational Performance Measure has been identified as a useful tool for measuring functional recovery among youth participating in active rehabilitation [7]. Concurrent measurement of symptoms and occupational performance can provide a more complete assessment of recovery than symptom reduction alone.

#### Factors That Cause and Sustain the Problem

Current evidence indicates that persistent symptoms and activity limitations after concussion are best understood through a biopsychosocial perspective [38,39,60]. A key tenet of the biopsychosocial model is that the interaction among biological, psychological, and social factors determines health outcomes and that these factors must therefore not be addressed in isolation [61].

Biological or physiological factors after concussion include autonomic nervous system and cerebral autoregulatory dysfunction [35], impaired balance and coordination [62], visual and vestibular dysfunction [63], sleep disturbances, and fatigue [64]. Exercise intolerance, which reflects autonomic and cerebrovascular dysfunction, is a prognosticator of recovery [65], as are visual and vestibular dysfunction [63].

There is growing recognition that psychological mechanisms related to cognitive, behavioral, and emotional reactions to concussion influence outcomes [39]. Negative illness perceptions, including beliefs about injury timelines, consequences, and level of control, are associated with poor outcomes [60,66]. Various cognitive biases may precipitate negative illness perceptions, including causal misattribution (ie, misattribution of common benign symptoms to concussion), catastrophizing (ie, misinterpretation of symptoms as dangerous), good-old-days bias (ie, tendency to overestimate preinjury health or function), and the nocebo effect (ie, concussion outcomes shaped by the expectations of illness or dysfunction after the injury) [39]. Negative illness perceptions often lead to maladaptive coping behaviors such as fear avoidance (ie, fear of provoking symptoms leads to avoidance of activities), endurance (ie, pushing through symptoms), or all-or-nothing behavior (ie, alternating between periods of extremely low and extremely high levels of activity) [60,67]. Emotional responses such as stress, anxiety, and depression can result from, or reinforce, negative perceptions and behaviors and have been shown to influence outcomes [68]. In youth, self-efficacy is also reduced after concussion [69], and lower levels of self-efficacy predict greater symptom burden [70]. Cognitive, behavioral, and emotional factors interact to form vicious cycles that maintain the experience of symptoms and functional limitation.

The social influences of concussion are poorly understood. Adolescents with concussion report limitations in school function and social activities [7-9]. Qualitative studies show that concussion has adverse effects on the interpersonal relationships of youth and that the influence of relationships on recovery can be positive or negative [8,71]. In the study by Kita et al [72], female adolescents with concussion identified their friends, parents, clinicians, and peers with personal histories of concussion as key providers of social support that mitigates various challenges in their recovery. Among collegiate athletes, greater satisfaction with social support has been associated with lower postconcussion anxiety [73].

Access to appropriate clinical care may influence how these biological, psychological, and social factors are experienced by the individual and, in turn, their effects on recovery. A systematic review found several studies showing that earlier initiation of clinical care after concussion leads to quicker recovery [74]. Timely access to care allows for early initiation of supervised subsymptom threshold aerobic exercise [33,34]. Conversely, those without guidance may continue to rest until achieving spontaneous symptom resolution, which increases their risk for physical deconditioning and mood disturbances [11,12] or for engaging in levels of activity that may be harmful for recovery. Early education and reassurance from a health care provider can reduce unfavorable psychological responses to injury [75], whereas poor access to care may itself contribute to negative injury perceptions. Communication with a health care provider can also build the capacity of youth to seek social support by providing information about appropriate school accommodations, helping youth to communicate the nature of their injury and encouraging youth to engage those around them to support their recovery [72].

There are currently significant challenges to accessing timely appropriate care for concussion in Canada [49]. Studies have identified knowledge gaps regarding concussion management among primary care providers [76,77]. Access to specialized concussion care is also limited in Canada, especially among individuals in remote communities who may experience significant geographic or socioeconomic barriers and youth who are reliant on caregiver accompaniment. Studies from the United States reveal barriers to accessing pediatric concussion care among families in rural communities, including higher health care expenses [78] and indirect costs related to transportation and lost productivity [79]. Delayed access to care is problematic because it increases the risk of premature return to activity and potential reinjury or development of persistent symptoms and functional limitation.

#### Identifying Similar Interventions

A critical review of the literature describing active rehabilitation approaches to concussion was performed in the fall of 2019. Key articles were located through search in Google Scholar and PubMed using keywords that included *active rehabilitation*, *exercise*, *physical activity*, *concussion*, *mild traumatic brain injury*, *youth*, and *adolescents*. Additional pertinent literature was identified through forward and backward searching of reference lists as well as literature previously known to the authors. Intervention characteristics among the studies included in the review are detailed in Multimedia Appendix 1 [7,16-21,24-30,32,80] and briefly summarized in the following paragraphs.

Findings from the review indicated that active rehabilitation interventions typically last 6 weeks and involve a variety of components, including aerobic exercise [7,16,19,20,24-30,32,80-84], sport coordination drills [25-30,32,80,82,83], balance training [32], visualization or relaxation [25-30,80-83], and education and support [25-30,80-83]. The 6-week timeline was established by Gagnon et al [29], who found that a mean intervention duration of 4.4 (SD 2.6) weeks was required to achieve symptom resolution. All the studies identified implemented individual interventions. Active rehabilitation has typically been evaluated through changes in postconcussion symptoms [16-18,24-30,32,82,84]. Improved mood [29,30,80], quality of life [17,30], and occupational performance [7] have also been reported.

Although key components are not fully understood, a study of multimodal active rehabilitation found that youth identified the education, aerobic exercise, and sport coordination drills as most helpful [81]. A combination of education regarding energy management and engagement in supervised physical activity is therefore essential to the approach [81]. Future iterations of active rehabilitation should consider a focus on self-management through education and supervised physical activity involving aerobic and coordination exercise.

Published active rehabilitation interventions typically involve a home exercise program performed several (3-7) days per week and weekly appointments with a clinician. Appointments have occasionally been conducted by telephone [17,31,32], which was reportedly appreciated by participants because the telephone appointments reduced travel requirements [17]. The identified studies required participants to attend in-person appointments for pre- and postintervention assessments, indicating an opportunity to explore interventions designed to be delivered entirely remotely and requiring no in-person appointments.

Systematic reviews of telerehabilitation [45-47] and remote self-management programs [85,86] were consulted to identify best practice recommendations regarding the remote service delivery model for the Tele-AR intervention. Consistent communication with a health care provider was identified as key to promoting retention and positive outcomes among remotely delivered self-management interventions [85]. Ongoing individualized education, lifestyle intervention, adherence support, and clinical review with feedback were also identified as important components [86].

The aforementioned literature provided the foundations for the design of the Tele-AR intervention. On the basis of this literature, it was determined that the intervention should be a 6-week program comprising aerobic exercise, coordination drills, and comprehensive individually tailored education and support to train self-management skills. Telehealth literature indicated that weekly appointments should involve a review of symptoms and activity performance with provider feedback, continued education regarding symptom management strategies, and support to promote motivation and adherence to the home program [85,86].

#### Defining Target Context

Tele-AR could be an accessible intervention to reduce the burden of pediatric concussion, especially among those identified as high risk for prolonged recovery [3]. It complements ongoing telemedicine initiatives to improve access to pediatric concussion care in rural and remote Canadian communities [48,49].

Recommendations for the use of telemedicine in concussion management specify the need for initial in-person medical assessment to confirm the diagnosis of concussion as well as regular medical follow-up [49,87]. Tele-AR is therefore designed to serve as an adjunct intervention for youth who have previously undergone in-person medical assessment and continue to receive medical follow-up, including appropriate referral for targeted treatments such as cervical or vestibular therapy. Although there is evidence that earlier initiation of supervised exercise (after 24-48 hours of initial rest) leads to quicker recovery [88], many without access to care continue to rest until symptom resolution, which may contribute to delayed recovery [12]. Remote delivery of active rehabilitation could increase access to care that enables earlier resumption of supervised physical activity and contributes to quicker recovery.

### Identifying and Applying Theory

#### Supporting Motivation During Active Rehabilitation

We drew on self-determination theory (SDT) to expand the theoretical foundation for active rehabilitation to address motivation, a key determinant of engagement and outcomes in rehabilitation [41,89]. Motivation is a key facilitator of participation after childhood traumatic brain injury [90] but is often reduced among individuals with a brain injury [42]. Existing theoretical underpinnings of active rehabilitation recognize the importance of motivation [28] but do not address how to promote motivation.

SDT has been identified as a useful framework for conceptualizing motivation in pediatric rehabilitation [41]. SDT is a theory of human motivation that describes the influence of social and cultural factors on an individual’s sense of volition (self-determination), performance, and well-being [91]. According to SDT, three psychological needs foster intrinsic motivation: competence (feeling mastery and success), autonomy (feeling of being able to choose one’s own actions), and relatedness (feeling positive relationships with others) [91]. Systematic reviews demonstrate that health interventions designed using SDT can increase satisfaction of psychological needs, leading to improvements in physical and mental health outcomes [91,92]. Previous articles have highlighted the utility of SDT to identify factors that influence motivation during brain injury rehabilitation [42]. In adults, mild traumatic brain injury reduces fulfillment of psychological needs, suggesting that rehabilitation should address these variables [93].

The Tele-AR intervention is designed to support the 3 psychological needs outlined in SDT to promote motivation and engagement in the program and regular daily activities. Each element is briefly described in the following sections. Table 1 provides examples of how the psychological needs are addressed in the Tele-AR intervention, using the SDT taxonomy from Teixeira et al [94].

**Table 1:** Self-determination theory (SDT) strategies in the Tele-Active Rehabilitation (Tele-AR) intervention.

SDT strategy		Application to Tele-AR intervention
**Autonomy support**		
	MBCT^a^ 1: Elicit perspectives on condition or behavior	Elicit perspectives of youth regarding their concussion and associated challenges Tailoring of education and support to address individual perspectives
	MBCT 3: Use noncontrolling, informational language	Provide education using a nonjudgmental approach that emphasizes freedom of choice
	MBCT 5: Providing meaningful rationale	Provide rationale for active rehabilitation approach, including the role of exercise to support recovery, energy management techniques, and gradual return to activity
	MBCT 6: Provide choice	Engage youth in coconstruction of the active rehabilitation program based on their individual needs and interests Empower youth to take responsibility for their own recovery through self-management skills and coping strategies
**Relatedness support**		
	MBCT 8: Acknowledge and respect perspectives and feelings	Acknowledge perspectives of youth regarding the active rehabilitation approach and intervention components
	MBCT 9: Encourage asking questions	Encourage questions from participants during weekly appointments
	MBCT 10: Show unconditional regard	Express empathy and provide positive support regardless of exercise completion
	MBCT 11: Demonstrate interest in the person	Demonstrate interest in activity interests and experience of youth Integrate participants’ needs and interests in home program
	MBCT 12: Use empathic listening	Demonstrate active listening by maintaining eye contact and head nods Provide meaningful summaries of comments by youth and check understanding
	MBCT 13: Provide opportunities for ongoing support	Provide telephone and email contact information for ongoing communication outside of regular appointments
	MBCT 14: Prompt identification and seek available social support	Encourage social support seeking from parents, teachers, coaches, and friends Teach metaphors to communicate the experience of having a concussion, such as comparing energy levels to a cellphone battery Inclusion of friends in exercise program when possible
**Competence support**		
	MBCT 15: Address obstacles to change	Prompt identification of potential barriers and solutions
	MBCT 17: Assist in setting optimal challenge	Review return to sport and return to school protocols Discuss appropriate academic accommodations for return to school Support in setting goals for gradual increase in exercise and activity engagement
	MBCT 18: Offer constructive, clear, and relevant feedback	Provide positive feedback and encouragement for successes with school, exercise, and other activities Discuss strategies used by other youth facing similar challenges
	MBCT 19: Help develop a clear and concrete plan of action	Prompt participants to develop personal concussion toolbox recovery plan describing which strategies they intend to use Provide clear instructions for home exercise program
	MBCT 20: Promote self-monitoring	Instruct to monitor perceived exertion and symptoms during exercise Prompt monitoring of activity duration to facilitate gradual progression Highlight progress toward exercise goals and activity resumption Draw attention to positive physiological and affective states after exercise
	MBCT 21: Explore ways of dealing with pressure	Discuss relaxation and coping strategies such as diaphragmatic breathing, progressive muscle relaxation, and visualization

^a^MBCT: motivation and behavior change technique (according to the self-determination theory taxonomy developed by Teixeira et al [94]).

#### Competence

The Tele-AR intervention aims to promote a sense of competence by helping adolescents set optimal challenges and increase self-efficacy. This involves gradually increasing experiences of success through participation in school, exercise, and other meaningful activities. An action plan is developed with each participant to identify how they will implement strategies to support recovery (eg, energy management and relaxation). Second, participants are taught to self-monitor exertion during activities to prevent significant symptom exacerbation. They are encouraged to monitor the time during which they engage in activities such as schoolwork and screen use to facilitate gradual progression. Third, the clinician provides feedback and encouragement during appointments using a strengths-based approach, whereby participants are prompted to discuss what has gone well and which strategies they used to support success. An opportunity is provided for participants to identify barriers they are encountering and explore potential solutions with the clinician. Finally, strategies are discussed for dealing with stressful situations that could undermine competence, such as relaxation techniques (eg, deep breathing, muscle relaxation, and visualization) and seeking help.

#### Autonomy

Autonomy is supported by providing meaningful rationales for active rehabilitation, integrating participant perspectives in program development, and providing choice. The rationale for the active rehabilitation approach is explained to participants at the beginning of the intervention. Education is provided using nonjudgmental language integrating participant perspectives and emphasizing freedom of choice (ie, which strategies to use and how to use them). Participants are engaged in coconstructing the home program based on their needs and interests, including choice about specific exercises. The Tele-AR intervention is designed to empower youth to take responsibility for their recovery through self-management and coping skills. In a previous study, parents reported that active rehabilitation helped their children become more accountable for their recovery by teaching them to self-monitor their condition [81]. Remote service delivery may further enhance the accountability of youth by eliminating the need for parental accompaniment to appointments.

#### Relatedness

Relatedness (ie, feeling positive relationships with others) is fostered by the clinician through active listening, expressing empathy, and encouraging perspectives and questions from the participant. Studies from a variety of rehabilitation disciplines, including physiotherapy [95], occupational therapy [96], and psychology [97], demonstrate that therapeutic alliance can be developed effectively through videoconferencing. Weekly appointments foster a strong therapeutic relationship, which has been shown to influence adherence to home exercise programs [98]. In addition, the clinician encourages social support seeking from parents, teachers, coaches, and friends using strategies identified by Kita et al [72]. For example, because of the invisible nature of concussion, some adolescents report being called “fakers” by peers [9]. Participants may therefore be taught metaphors to help communicate their *invisible* injury, such as comparing energy levels to a cellphone battery [72]. Participants are also encouraged to involve friends in the exercise program where possible, such as having a friend join their walk or including a teammate in sport-specific coordination drills.

### Modeling Process and Outcomes

**Figure 1:**
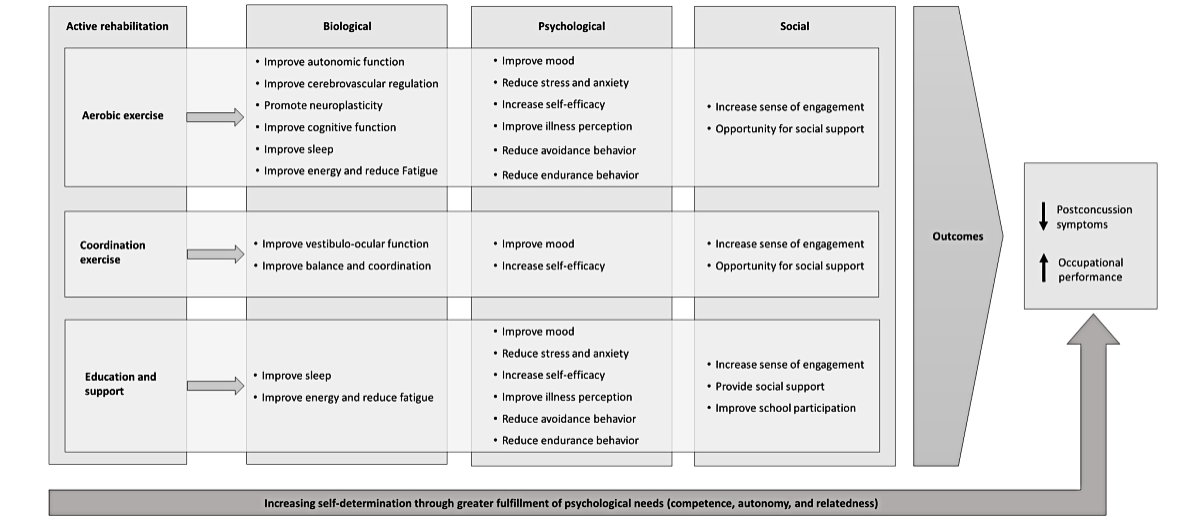
Logic model for the Tele-Active Rehabilitation intervention.

Modeling aims to define intervention components, explain how they relate to each other, and describe the mechanism through which they influence desired intervention outcomes [51]. Figure 1 presents a logic model for the Tele-AR intervention. It describes the predicted effects of intervention components (ie, aerobic exercise, coordination drills, and education and support) based on the review by Gagnon et al [28] and published biopsychosocial models of concussion [38,39]. Descriptions of anticipated outcomes from each intervention component are provided in the following paragraphs.

Several reviews demonstrate the efficacy of aerobic exercise for reducing postconcussion symptoms [22,23]. Progressive subsymptom threshold aerobic exercise improves autonomic function and cerebral blood flow regulation, which are impaired after concussion [33-35]. Aerobic activity also facilitates neurological recovery by promoting neuroplasticity [99]. Cardiovascular fitness improves with aerobic training, which can reduce fatigue and improve energy levels [100]. The psychological benefits of aerobic activity include stress reduction, elevated mood, and increased self-efficacy [101]. Supervised exercise may also reduce avoidance and endurance behaviors, both associated with poor outcomes [67]. Graded and supervised subsymptom threshold aerobic exercise builds mastery experiences that challenge catastrophic assumptions about symptoms and activity, which may improve illness perception and reduce activity avoidance [17]. Teaching those who display endurance behavior to monitor exertion during exercise also supports pacing strategies that facilitate activity performance [15]. For individuals who are experiencing restrictions after concussion, exercise can provide a sense of engagement in meaningful activity and create opportunity for social connection [38,81].

According to Gagnon et al [28], the purpose of coordination exercises is to continue light exercise and reintroduce familiar activities in a successful context to improve mood and increase self-efficacy. Allowing choice in coordination exercises enables youth to participate in familiar meaningful activities. A previous study found that youth enjoy the coordination drills because they allow for gradual reintroduction of meaningful activities and help improve overall fitness [81]. These exercises may also target deficits in balance, coordination, and visual function that may occur after concussion [2]. Finally, sport-specific coordination training provides an opportunity for social connection because participants may complete the drills with a friend or teammate.

Education and support are essential components of concussion management consistently recommended among evidence-based guidelines [102]. Education about the nature of concussion and recovery timelines addresses important psychological variables that predict recovery, such as illness perception [66]. Energy management and sleep hygiene strategies may reduce fatigue and improve energy levels [81]. A cohort of youth who participated in active rehabilitation identified energy management as a key component, which parents believed improved their child’s self-management [81]. Providing support and training in coping strategies may also reduce anxiety and emotional distress, which are common after concussion and predict poor outcomes [66]. Helping youth to identify appropriate accommodations and return-to-learn strategies can improve school participation and has been identified by families as a priority service need [103]. Families may also require assistance modifying other activities to support a gradual return, such as playing an instrument or spending time with friends [7]. Ultimately, education and support may increase self-efficacy for activity performance after concussion, which has been associated with lower symptom severity in children and adolescents [70].

As described in the *Identifying and Applying Theory* section, the Tele-AR intervention is designed to foster satisfaction regarding competence, autonomy, and relatedness to strengthen motivation and self-determination. Fulfillment of these needs is believed to support participation in the program. It is also hypothesized that increased self-determination through active rehabilitation will directly contribute to symptom reduction and improved occupational performance.

### Tele-AR Intervention

#### Intervention Overview

The Tele-AR intervention is a 6-week home program facilitated through weekly videoconferencing appointments with a rehabilitation clinician (kinesiologist, occupational therapist, or physical therapist). It consists of (1) aerobic exercise, (2) sport coordination drills, and (3) comprehensive concussion education and support. The following sections provide a detailed description of the intervention.

#### Intervention Timeline

**Figure 2:**
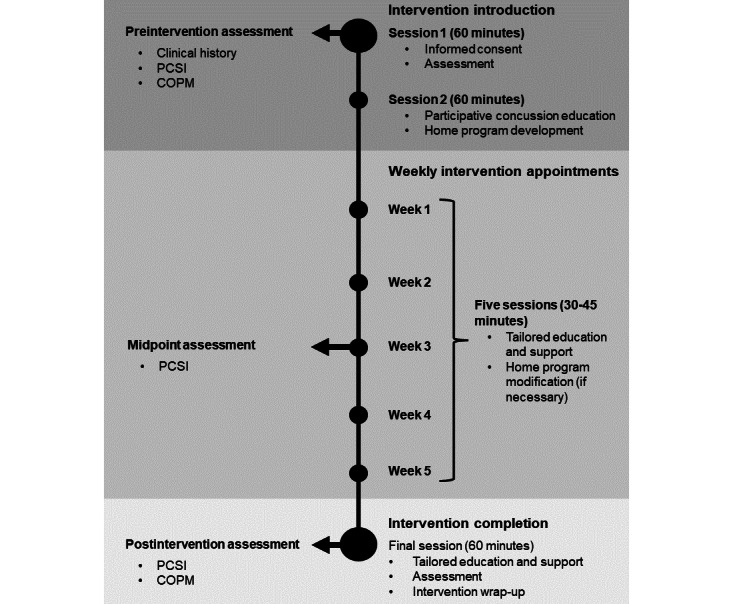
Overview of the Tele-Active Rehabilitation intervention. COPM: Canadian Occupational Performance Measure; PCSI: Postconcussion Symptom Inventory.

Figure 2 presents an overview of the timeline for the Tele-AR intervention. It begins with assessment and program introduction spread over 2 appointments, each lasting for 1 hour. The first appointment is dedicated to completing the informed consent procedures for the research study and preintervention assessments (see the *Assessment* section). The second appointment consists of a participative concussion education session and collaborative coconstruction of the home program, integrating the participant’s individual needs and interests within the three components (aerobics, coordination drills, and education and support).

During weeks 1 to 6, participants are asked to perform the home exercise program 3 days per week and meet with the research clinician once per week. The purpose of weekly appointments is to provide continued education and support, modify the exercise program as appropriate, and strengthen motivation for engaging in the home program and other daily activities. The week 3 appointment includes symptom reassessment to evaluate interim changes. Significant deterioration at this point could indicate a need for in-person reassessment or referral to other providers [87]. Postintervention assessment occurs in week 6.

#### Assessment

Preintervention assessment is conducted through a clinical interview with youth and their parents during the first appointment and is used to inform home program prescription. Assessment is repeated in week 6 to evaluate postintervention changes.

Assessment is intentionally targeted to ensure clinical application. It includes clinical history of injury details and factors known to affect recovery (eg, concussion history, medical and mental health history, and social history [3,58]), self-reported symptom rating (Postconcussion Symptom Inventory [59]), and self-perceived occupational performance, a measure of performance in daily activities (Canadian Occupational Performance Measure [104]). These measures reflect current understanding of concussion recovery and call for patient-reported outcome measures in concussion rehabilitation that focus on function and well-being [13,105].

#### Intervention Components

##### Overview

The Tele-AR intervention comprises the following components: (1) aerobic exercise, (2) sport coordination drills, and (3) comprehensive concussion education and support. Components are individually tailored to the specific needs of each participant. The exercise program is prescribed for 3 days per week to promote compliance by minimizing disruption to daily activities and to allow for comparison between days with and without exercise. Imhoff et al [32] showed that 3 sessions per week is sufficient to facilitate symptom reduction and was associated with high treatment adherence. A description of each component is provided in the following sections.

##### Aerobic Exercise

After initial assessment, each participant is prescribed aerobic exercise beginning with 10 minutes of low-intensity activity at level 4 (*Just feeling a strain*) on the Pictorial Children’s Effort Rating Table (PCERT) [106]. Aerobic exercise may be completed on an exercise machine (treadmill, stationary bike, or elliptical trainer), indoor track, or outdoor space, depending on resources available. Participants are instructed to terminate exercise and rest upon symptom exacerbation (see *Safety* section); the time to symptom exacerbation becomes the new target duration. Exercise duration and intensity are progressed during weekly appointments if symptoms are well tolerated, following the standardized protocol presented in Table 2.

The initial aerobic prescription (10 minutes, PCERT level 4) is based on the time to symptom exacerbation reported by Gagnon et al [28] and Dobney et al [26] among youth initiating active rehabilitation. This prescription is consistent with other study protocols [31,107], as well as recommendations from literature reviews and clinical guidelines for exercise after concussion [108,109]. Previous studies demonstrate the utility of perceived exertion as a method for prescribing exercise to youth with concussion [32,110]. The PCERT is a validated tool for assessing effort perception in youth [106] and has been used previously to facilitate the home program in active rehabilitation [26,29-31].

**Table 2:** Aerobic exercise progression^a^ protocol.

Week	Duration (minutes)	Intensity (PCERT^b^ level)
1	10	4
2	15	4
3	15	5
4	20	5
5	20	6
6	30	6

^a^Progression was only recommended if significant symptom exacerbation did not occur in the previous week.

^b^PCERT: Pictorial Children’s Effort Rating Table.

##### Coordination Drills

Coordination drills are individualized based on participant interests and preintervention assessments. They may target balance, coordination, sport-specific skills, or general health exercises. For example, a basketball player may be given shooting drills first performed stationary and progressed to include dynamic movement. Coordination exercises are performed for up to 10 minutes at the same intensity as the aerobic component and are also terminated at the onset of any new or worsening symptoms. Participants are provided with written instructions outlining the details of their home exercise program.

##### Education and Support

Individualized education and support regarding symptom management and return to activity are provided to participants throughout the intervention. The education curriculum is based on the evidence-supported *Concussion & You* self-management program [111], which covers topics that include energy management, relaxation, nutrition, hydration, sleep hygiene, and return to school and sport. Education material is delivered through a participative session and reviewed weekly with specific application to challenges experienced by participants in their daily life. Participants are also provided with the *Concussion & You* education handbook [112] to consolidate information.

#### Technology

Appointments in the Tele-AR intervention are conducted through real-time videoconferencing using a secure platform that meets health privacy standards and allows for collaboration features such as screen sharing. The clinician uses a standard laptop or desktop computer, and participants may engage in the videoconferencing appointments using a computer, tablet, or smartphone.

Videoconferencing is believed to facilitate therapeutic rapport and is the most common method of communication in telerehabilitation for children [46]. Mental health support for youth is acceptable and effective when delivered through videoconferencing [113], and a study in adolescents with concussion found similar ratings of therapeutic alliance and satisfaction between face-to-face and video telehealth visits [114]. Although some individuals with concussion experience screen sensitivity, current evidence does not indicate that strict avoidance of screens improves recovery [115], supporting instead an individualized approach to screen use within tolerable limits. Other screen-based interventions for concussion have been tolerated well, with no screen sensitivity issues reported [116,117]. Subsequent feasibility testing of this intervention will assess participant perceptions and experiences engaging in video-based appointments.

#### Safety

Special attention must be paid to ensure safety in telehealth interventions. Safety considerations for the Tele-AR intervention are summarized in Textbox 2.

Education regarding activity-related symptom exacerbation and the appropriate response is provided during program prescription. Evidence suggests that activity-related symptom exacerbations are transient and not detrimental to recovery [118]. Exercise-related symptom exacerbation generally resolves within 1 hour of rest [17,25,26]. Participants are instructed to terminate exercise upon significant symptom exacerbation, which is operationalized according to the definition from Leddy et al [119] as an increase of ≥3 points on a 10-point visual analog scale (VAS) [119]. Participants are familiarized with the Wong-Baker FACES pain rating VAS for symptoms [119]. The VAS is used by participants to rate their current overall symptom experience before exercise and monitor for any increase during the activity. Participants are instructed to contact the research team if any of the following occurs: (1) exercise-induced symptom exacerbation that does not subside within 24 hours of rest, (2) exercise-induced symptom exacerbation experienced during consecutive exercise sessions, and (3) the participant has concerns about exercise. In these scenarios, the research team provides appropriate support, discusses potential exercise program modification, and considers referral to the primary care provider or other providers. The stopping rule described by Dobney et al [26] is used, whereby the exercise program is suspended if symptom exacerbation occurs during 3 consecutive sessions despite exercise modification.

**Safety considerations for the Tele-Active Rehabilitation intervention.**

**Symptom exacerbation considerations**

- Education regarding activity-related symptom exacerbation and appropriate response
- Participants instructed to terminate exercise and rest upon significant symptom exacerbation (an increase of ≥3 points on a 10-point visual analog scale)
- Suspension of exercise program pending medical clearance if symptom exacerbation occurs during 3 consecutive sessions despite exercise modification
- Participants instructed to contact the research team in the event of exercise-induced symptom exacerbation that does not subside within 24 hours of rest or exercise-induced symptom exacerbation during consecutive exercise sessions

**Exercise safety considerations**

- Preparticipation screening of contraindications to exercise
- Ensuring an open space for exercise free from tripping hazards
- Advising on use of support for balance (if needed)
- Considering parental supervision during exercise sessions

**General telehealth considerations**

- Confirming participant location, emergency contact information, and alternative methods of communication before appointment commencement
- Development of safety protocol in case of acute medical or mental health emergency

## Discussion

### Contributions

In this paper, we describe the development and theoretical foundation of the Tele-AR intervention for youth with concussion, specifically designed for remote service delivery. Development was guided by the MRC framework for complex interventions, involving the integration of existing evidence with appropriate theory [50]. Although growing evidence supports an active approach to concussion rehabilitation involving closely monitored progressive exercise combined with education and support, families often face several barriers that limit engagement in such care. The Tele-AR intervention was created to address these barriers. Intervention development is an iterative process, and results from an ongoing mixed methods feasibility study will inform further improvements.

The Tele-AR intervention builds on previous work advocating for a holistic approach to concussion care that emphasizes function and general well-being [13]. It is among the first interventions designed to improve activity and participation among youth with concussion [120]. Components are designed to concurrently address biological, psychological, and social aspects of concussion in a remote service delivery format. We emphasize education and support as essential to the intervention and expand the notion of active rehabilitation to facilitate motivation and performance in activities that are meaningful to the individual.

### Further Study

The logic model presented here requires further study. Evaluating changes in identified biopsychosocial constructs may improve clinical delivery of active rehabilitation. Continued efforts to reconceptualize recovery from concussion through a biopsychosocial lens with an emphasis on function and participation may also identify new targets for change in rehabilitation and inform a stronger theoretical framework [13]. The qualitative exploration of youth and parent perspectives regarding perceived needs from the Tele-AR intervention is addressed in the feasibility study, and it will help refine intervention components and delivery.

The development process involved extensive review of the literature to integrate current evidence. However, the literature review methodology was not systematic and thus may be subject to selection bias or missed publications. In addition, most of the literature was reviewed before the COVID-19 pandemic when telehealth use and scholarship rapidly increased. Given the breadth of active rehabilitation approaches described in the literature and rapid growth of research in this field, future work using a more intentional review methodology and original investigation is warranted to determine optimal intervention approaches. All studies identified in the review implemented individual interventions. Group interventions may foster a greater sense of relatedness and social support and should be explored in this population.

### Conclusions

Tele-AR complements ongoing telemedicine initiatives to improve access to concussion care [49] and may represent an accessible proactive intervention for those identified as high risk for prolonged recovery. The literature reviewed here may also be helpful to clinicians and families of youth with concussion to inform remote care during the COVID-19 pandemic. Research to evaluate the feasibility of this intervention is underway, and if warranted, more rigorous study should be undertaken to determine intervention effects and contribute to identifying best practices for telehealth concussion services that support youth in a timely return to activity.

## Appendix

Multimedia Appendix 1 Intervention characteristics among studies included in the review.

## Abbreviations

MRC: Medical Research Council
PCERT: Pictorial Children’s Effort Rating Table
SDT: self-determination theory
Tele-AR: Tele-Active Rehabilitation
VAS: visual analog scale
