# Supplementary material for: Tele-Active Rehabilitation for Youth With Concussion: Evidence-Based and Theory-Informed Intervention Development
Source: JMIR Pediatr Parent. 2022 Apr 4;5(2):e34822. doi: 10.2196/34822 (PMC9016504; doi:10.2196/34822)
Supplement: Multimedia Appendix 1 [file pediatrics_v5i2e34822_app1.pdf]

Supplementary Table 1. Intervention characteristics among studies included in review

First author, year [reference]	Study design	Participants (sample size)	Intervention details: Duration, components, training frequency, supervision	Key outcome measures
Sang, 2019 [7]	Prospective quasi-experimental repeated measures design	Youth ages 9-18 years with post-concussion symptoms at least 2 weeks post-injury (n=52)	• Duration: 6 weeks.<br>• Components: Comprehensive education, graded low-intensity aerobic exercise (walking or stationary bike) at 50-60% HR_{max} or level 4/10 on Pictorial Children's Effort Rating Table (15 minutes), sport-specific coordination drills (10 minutes), and relaxation exercises (5-10 minutes).<br>• Training frequency: 7 days/week.<br>• Supervision: In-person assessment at 0, 3, and 6. Phone check-ins at week 1, 2 and 4.	• Post-concussion symptoms (Post-Concussion Symptom Inventory)<br>• Occupational performance and satisfaction (Canadian Occupational Performance Measure)
Kurowski, 2017 [16]	Randomized clinical trial	Adolescents ages 12-17 years with persistent post-concussion symptoms 4-16 weeks post-injury (n=30)	• Duration: 6 weeks.<br>• Components: Progressive aerobic exercise (stationary cycling) at Borg RPE intensity level 11-16.<br>• Training frequency: 5-6 days/week.<br>• Supervision: Weekly in-person assessment.	• Post-concussion symptoms (Post-Concussion Symptom Inventory)
Chrisman, 2019 [17]	Pilot randomized control trial	Adolescents ages 12-17 years with persistent post-concussion symptoms (n=30)	• Duration: 6 weeks.<br>• Components: Progressive aerobic exercise training based on results from Buffalo Concussion Treadmill Test.<br>• Training frequency: 7 days/week.<br>• Supervision: In-person assessment at weeks 0 and 6. Weekly telephone calls weeks 1-5.	• Post-concussion symptoms (Health Behaviour Inventory)
Chrisman, 2017 [18]	Retrospective cohort study	Youth ages 12-18 with persistent post-concussion symptoms >1 month post-injury (n=83)	• Duration: Until symptom resolution.<br>• Components: Progressive aerobic exercise training based on results from Buffalo Concussion Treadmill Test.<br>• Training frequency: 7 days/week.<br>• Supervision: In-person assessment every 1-2 weeks.	• Post-concussion symptoms (SCAT-2)
Leddy, 2019 [19]	Multicentre randomized clinical trial	Adolescent athletes ages 13-18 years presenting within 10	• Duration: Until symptom resolution.<br>• Components: Progressive aerobic exercise training based on results from Buffalo Concussion Treadmill Test.	• Post-concussion symptoms (Post-Concussion Symptom Scale)

		days of sport-related concussion (n=103).	• Training frequency: 7 days/week.<br>• Supervision: Weekly in-person assessment	
Leddy, 2019 [20]	Quasi-experimental Trial	Adolescent athletes ages 13-18 years presenting within 1-9 days of sport-related concussion (n=54).	• Duration: Until symptom resolution.<br>• Components: Progressive aerobic exercise training based on results from Buffalo Concussion Treadmill Test.<br>• Training frequency: 7 days/week.<br>• Supervision: Weekly in-person assessment	• Post-concussion symptoms (Post-Concussion Symptom Scale)
Leddy, 2010 [21]	Prospective case series	Youth and adult athletes 16-53 years old experiencing persistent post-concussion symptoms (n=12)	• Duration: Until symptom resolution.<br>• Components: Progressive aerobic exercise training based on results from Buffalo Concussion Treadmill Test.<br>• Training frequency: 5-6 days/week.<br>• Supervision: In-person assessment every 3 weeks.	• Post-concussion symptoms (Graded Symptom Checklist)
Willer, 2019 [24]	Quasi-experimental Trial	Adolescent athletes aged 13-18 years presenting within 10 days of sport-related concussion (n=103)	• Duration: Until symptom resolution.<br>• Components: Progressive aerobic exercise training based on results from Buffalo Concussion Treadmill Test.<br>• Training frequency: 7 days/week.<br>• Supervision: Weekly in-person assessment	• Post-concussion symptoms (Post-Concussion Symptom Scale)
Chan, 2018 [25]	Randomized clinical trial	Youth ages 12-18 with persistent post-concussion symptoms >1 month post-injury (n=19)	• Duration: 6 weeks.<br>• Components: Comprehensive education, graded low-intensity aerobic exercise at 50-60% HR_{max} or level 4/10 on Pictorial Children's Effort Rating Table (15 minutes), sport-specific coordination drills (10 minutes), and relaxation/visualization exercises (5 minutes).<br>• Training frequency: 7 days/week.<br>• Supervision: Weekly in-person reassessment.	• Post-concussion symptoms (Post-Concussion Symptom Scale)
Dobney, 2019 [26]	Randomized clinical trial	Youth ages 9-17 years old with post-concussion symptoms at least 2 weeks post-injury (n=20)	• Duration: 6 weeks.<br>• Components: Comprehensive education, graded low-intensity aerobic exercise at 50-60% HR_{max} or level 4/10 on Pictorial Children's Effort Rating Table (15 minutes), sport-specific coordination drills (10 minutes), and relaxation/visualization exercises (5-10 minutes).	• Post-concussion symptoms (Post-Concussion Symptom Inventory)

			• Training frequency: 7 days/week.<br>• Supervision: Weekly in-person reassessment.	
Dobney, 2017 [27]	Retrospective analysis of prospectively collected clinical data	Youth ages 11-17 years old with post-concussion symptoms at least 3-4 weeks post-injury (n=277)	• Duration: 6 weeks.<br>• Components: Comprehensive education, graded low-intensity aerobic exercise at 50-60% HR_{max} or level 4/10 on Pictorial Children's Effort Rating Table (15 minutes), sport-specific coordination drills (10 minutes), and relaxation/visualization exercises (5-10 minutes).<br>• Training frequency: 7 days/week.<br>• Supervision: Weekly in-person reassessment.	• Post-concussion symptoms (Post-Concussion Symptom Scale)
Gagnon, 2009 [28]	Case series	Youth ages 10-17 years old with post-concussion symptoms at least 4 weeks post-injury (n=16)	• Duration: Until clinical recovery (mean 4.4 weeks, SD = 2.6 weeks)<br>• Components: Comprehensive education, graded low-intensity aerobic exercise at 50-60% HR_{max} or level 4/10 on Pictorial Children's Effort Rating Table (15 minutes), sport-specific coordination drills (10 minutes), and relaxation/visualization exercises (5-10 minutes).<br>• Training frequency: 7 days/week.<br>• Supervision: Weekly in-person reassessment.	• Post-concussion symptoms (Post-Concussion Scale-Revised)
Gagnon, 2016 [29]	Case series	Adolescents ages 14-18 years old with post-concussion symptoms at least 4 weeks post-injury (n=10)	• Duration: 6 weeks.<br>• Components: Comprehensive education, graded low-intensity aerobic exercise at 50-60% HR_{max} or level 4/10 on Pictorial Children's Effort Rating Table (15 minutes), sport-specific coordination drills (10 minutes), and relaxation/visualization exercises (5-10 minutes).<br>• Training frequency: 7 days/week.<br>• Supervision: Weekly in-person reassessment.	• Post-concussion symptoms (Post-Concussion Scale)<br>• Mood (Beck Depression Inventory – Second Edition)<br>• Energy level (Pediatric Quality of Life Multidimensional Fatigue Scale)
Gauvin-Lepage, 2018 [30]	Multicenter prospective quasi-experimental study	Youth ages 8-17 years old with post-concussion symptoms at least 4 weeks post-injury (n=49)	• Duration: 6 weeks.<br>• Components: Comprehensive education, graded low-intensity aerobic exercise at 50-60% HR_{max} or level 4/10 on Pictorial Children's Effort Rating Table (15 minutes), sport-specific coordination drills (10	• Post-concussion symptoms (Post-Concussion Symptom Inventory)<br>• Mood and anxiety (Beck Youth Inventory, Child Behaviour Checklist)

			minutes), and relaxation/visualization exercises (5-10 minutes).<br>• Training frequency: 7 days/week.<br>• Supervision: Weekly in-person reassessment.	• Energy level (Pediatric Quality of Life Multidimensional Fatigue Scale)<br>• Quality of life (Pediatric Quality of Life – Generic Module)
Imhoff, 2016 [32]	Case Series	Youth ages 10-17 years old with post-concussion symptoms at least 4 weeks post-injury (n=15)	• Duration: Until clinical recovery (mean duration: 49 ± 17 days).<br>• Components: Progressive low- to high-intensity aerobic exercise (20 minutes) at Borg RPE levels 2-5/10, sport-specific coordination exercises (10 minutes), and therapeutic balance exercises.<br>• Training frequency: 3 days/week.<br>• Supervision: Initial in-person assessment and weekly phone check-in.	• Post-concussion symptoms (Post-Concussion Symptom Inventory)
Hunt, 2020 [80]	Secondary analysis of prospective quasi-experimental repeated measures design	Youth ages 9-18 years with post-concussion symptoms at least 2 weeks post-injury (n=40)	• Duration: 6 weeks.<br>• Components: Comprehensive education, graded low-intensity aerobic exercise (walking or stationary bike) at 50-60% HR_{max} or level 4/10 on Pictorial Children's Effort Rating Table (15 minutes), sport-specific coordination drills (10 minutes), and relaxation exercises (5-10 minutes).<br>• Training frequency: 7 days/week.<br>• Supervision: In-person assessment at 0, 3, and 6. Phone check-ins at week 1, 2 and 4.	• Emotional and social well-being (Beck Youth Inventories, Second Edition)<br>• Parental reported psychological well-being (Child Behaviour Checklist)
